# A case of perianal condylomata acuminata in a pregnant woman

**DOI:** 10.11604/pamj.2025.52.164.50331

**Published:** 2025-12-17

**Authors:** Qi He, Jianping Zhou

**Affiliations:** 1 First People's Hospital of Linping District, Hangzhou, China

**Keywords:** Pregnant women, condylomata acuminata, cryotherapy

## Image in medicine

We report a case of perianal condylomata acuminata in a pregnant woman. The patient is a 28-year-old female. During a prenatal checkup in the third trimester of pregnancy, a large cauliflower structure was found around the anus. She then visited the dermatology department of our hospital. The patient's syphilis testing and HIV testing were both negative. Samples collected from genital lesions were positive for HPV types 6 and 11. Biopsy pathology suggests condylomata acuminata. Based on these examinations, we ultimately diagnosed it as perianal condylomata acuminata. We provided the patient with a cryotherapy treatment. Two weeks later, when the patient returned for a follow-up visit, it was observed that the perianal warts had increased in size compared to before. The patient refused to continue cryotherapy. The patient delivered a baby by cesarean section at 37 weeks without any complications. After delivery, our department provided three sessions of laser combined with photodynamic therapy, followed by regular follow-ups, and no recurrence was observed. We followed up on the newborn delivered by the patient before the age of one and found no warts.

**Figure 1:**
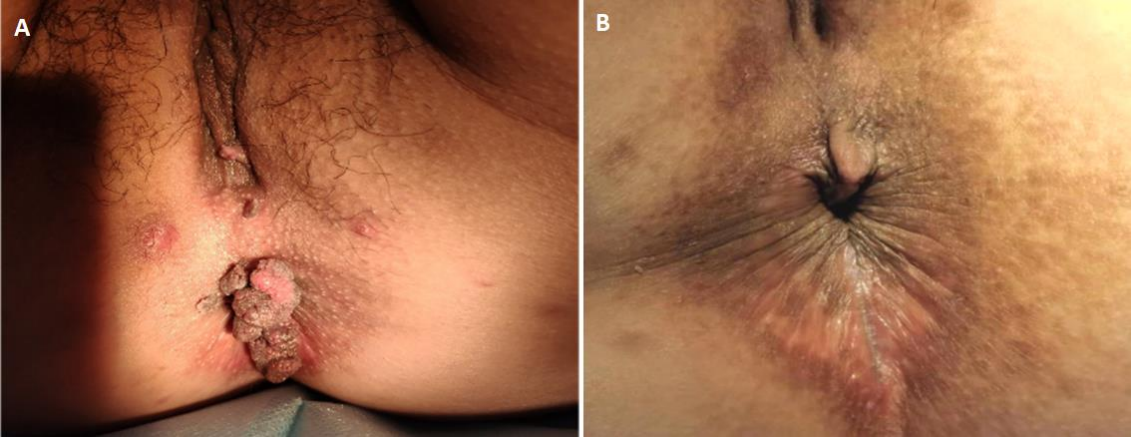
A) a large cauliflower structure around the anus; B) perianal skin after treatment

